# Epidemiological and genetic characterization of the influenza A (H1N1) virus in Hangzhou City in 2023

**DOI:** 10.3389/fpubh.2024.1464435

**Published:** 2024-11-20

**Authors:** Ningning Wang, Wei Lu, Li Yan, Mengru Liu, Feihu Che, Yue Wang, Chunli Yang, Mengyu Lv, Jun Cheng, Qingyang Sun, Yuzhu Dai

**Affiliations:** ^1^School of Laboratory Medicine, Bengbu Medical University, Bengbu, China; ^2^Department of Clinical Research, The 903rd Hospital of PLA, Hangzhou, China; ^3^Quality Control Division, The 903rd Hospital of PLA, Hangzhou, China; ^4^Military Casualty Management Section, The 903rd Hospital of PLA, Hangzhou, China

**Keywords:** influenza A virus, H1N1, epidemiology, genetic characterization, evolution

## Abstract

**Objective:**

To explore and describe the epidemiological and genetic variation characteristics of the influenza A (H1N1) virus in Hangzhou City.

**Methods:**

Respiratory throat swab specimens collected from the fever clinic of the 903rd Hospital of the Chinese People’s Liberation Army (PLA) between January and March 2023 were collected. The respiratory pathogen antigens were identified using the colloidal gold method, and those testing positive for influenza A virus antigens were confirmed and subtyped by RT-qPCR. Seventeen H1N1 isolates were selected to amplify hemagglutinin (HA) and neuraminidase (NA) gene sequences via RT-PCR, and sequencing was completed following the identification of the amplified products. The sequenced HA and NA sequences were spliced using DNASTAR software (version 5.0), and a phylogenetic tree was constructed using MEGA software (version 11.0) for genetic characterization.

**Results:**

A total of 2,376 respiratory samples were tested, with 680 cases testing positive for influenza A. Of these, 129 positive cases of influenza A were randomly selected for typing, resulting in the isolation of 112 H1N1 subtypes and 17 H3N2 subtypes. The HA genes of 17 strains of influenza A (H1N1) were randomly selected for amino acid homology comparisons with two vaccine strains recommended by the WHO for 2023 (A/Wisconsin/67/2022 (H1N1) and A/Victoria/4897/2022 (H1N1)). The HA gene results showed identities of 98.24 to 98.65% and 98.41 to 98.82%, respectively, and the NA gene results were 98.79 to 99.15% and 98.94 to 99.29%, respectively. Fourteen amino acid sites were altered in the HA gene of the 17 strains, with some strains contributing to the Sa and Ca antigenic determinants, respectively. Seventeen strains had mutations in the NA gene at sites 13, 50, 200, 339, 382, and 469. The sequenced strains, vaccine strains, and some 2023 domestic representative strains independently formed a branch 6B.1A.5a.2a.

**Conclusion:**

The continuous evolutionary mutations of the H1N1 virus genes in Hangzhou City suggest the possibility of the virus escaping from the immune response. This study provides an experimental basis for evaluating the protective effect of the vaccine and formulating preventive measures against influenza in Hangzhou City.

## 1 Introduction

Influenza A virus constitutes a negative-sense, single-stranded RNA virus that belongs to the Orthomyxoviridae family. It is categorized into various subtypes based on its surface glycoproteins, hemagglutinin (HA) and neuraminidase (NA) (1). There are 18 HA subtypes and 11 NA subtypes, which intercombine to generate diverse strains of influenza A viruses, each boasting distinct antigenic characteristics and pathogenic potentials. The influenza A (H1N1pdm09) pandemic initially emerged in Mexico and the United States in 2009. The first imported case of influenza A (H1N1pdm09) from the United States was documented in Sichuan, China, in May of that year (2). The virus was identified as a triple-recombinant virus, originating from a fusion of a swine influenza virus, an avian influenza virus, and a human influenza virus H1N1 (3). This recombination event gave rise to novel hybrid strains that triggered the influenza pandemic. These strains subsequently evolved into seasonal viruses and co-circulated with other seasonal influenza strains. As reported by the World Health Organization, the influenza pandemic swiftly propagated to 214 countries and regions, causing approximately 200,000 fatalities (4). Currently, the annual global mortality toll associated with seasonal influenza is estimated to range between 290,000 to 650,000 deaths, representing a substantial threat to public health security on a global scale (5, 6).

Influenza viruses facilitate broad-scale dissemination within populations by modifying their antigenic constituents through continuous antigenic drift, thereby eluding the host-derived immune response. Extensive research has corroborated the emergence of amino acid substitutions within the antigenic determinants of the hemagglutinin (HA) and neuraminidase (NA) genes, indicative of the viruses’ persistent evolutionary trajectory (7–9). Contemporary studies have intensified efforts to decipher the genetic diversity and permutations within the HA and NA genes, as well as their concomitant antigenic properties. Specifically, the HA protein is pivotal for the influenza A virus’s host cell invasion, with its gene being the most susceptible to mutation, particularly in the heavy chain domain of the HA protein which encompasses the antigenic and receptor binding sites (HA1). This domain is of paramount importance as it enables the virus to interact with the host cell’s surface receptors, facilitating viral uptake and playing a pivotal role in the infection and replication cascade. The NA protein, conversely, is responsible for the progeny virus particle release from the infected cell’s surface and the subsequent viral spread to adjacent cells, making it a prime target for anti-influenza therapeutics (10, 11). So far, there are three types of antiviral drugs approved for the treatment of influenza A virus infection (12), including transmembrane protein M2 ion channel inhibitors, neuraminidase inhibitors (NAIs), and polymerase inhibitors. Among them, M2 ion channel inhibitor amantadine is no longer recommended because of extensive drug resistance (13). Due to the widespread use of NAIs, the amino acids around the active site of NA have mutated, which also reduces the affinity of NA inhibitors for NA and leads to the emergence of drug-resistant strains (14). Presently, NA dimer inhibitors developed by researchers have been shown to have high NA inhibitory activity. Influenza virus polymerase, as the core machine of virus replication, is considered to be the most promising anti-influenza drug target at present, and inhibitors targeting different subunits of the polymerase are under clinical investigation. Consequently, a comprehensive analysis of the HA and NA genetic sequences is imperative for deciphering the evolutionary mechanisms underpinning H1N1 influenza viruses.

In the present study, we conducted a comprehensive analysis of influenza surveillance data compiled from respiratory illness cases attending the 903rd Hospital of the Chinese People’s Liberation Army (PLA) in the period from January to March 2023. Our investigation focused on characterizing the hemagglutinin (HA) and neuraminidase (NA) gene sequences of influenza A (H1N1) variants, aiming to elucidate the molecular dynamics of the influenza A virus H1N1 in Hangzhou. The objective of this research is to furnish critical insights into the efficacy of influenza vaccines, as well as to monitor the mutation patterns among the viral strains, thereby providing a scientific foundation for the prophylaxis and management of future influenza pandemics.

## 2 Materials and methods

### 2.1 Samples

From January to March 2023, a comprehensive collection of 2,376 respiratory specimens was conducted within the fever clinic of the 903rd Hospital of the People’s Liberation Army (PLA). Each specimen was ethically approved with the approval number 20240708/18/01/001, granted by the hospital’s Medical Ethics Committee, and preserved at −80°C within a refrigeration unit.

### 2.2 Methods

#### 2.2.1 Respiratory virus testing

The colloidal gold-based *in vitro* diagnostic assay, provided by Hangzhou Genesis Biodetection & Biocontrol Co., Ltd., was utilized to detect the antigens of influenza A virus, influenza B virus, adenovirus, syncytial virus, and *Mycoplasma pneumoniae* in clinical specimens from a total of 2,376 patients. The assay was conducted and the results were interpreted in strict accordance with the manufacturer’s instructions.

#### 2.2.2 Extraction of influenza a virus RNA

Viral RNA extraction was performed based on the principle of magnetic bead adsorption, utilizing nucleic acid extraction or purification reagents, and the Natch 96 nucleic acid extractor from Sansure Biotech Inc. All procedures were followed in strict accordance with the manufacturer’s instructions.

#### 2.2.3 Influenza a typing test

A total of 129 specimens from influenza A-positive cases were randomly selected for influenza typing (H1N1, H3N2, H5N1, H7N9) using the HiScript II One Step qRT-PCR Probe Kit, with primers provided by Beijing Tsingke Biotech Co. Ltd. (Table 1). Influenza A virus typing was conducted using an Applied Biosystems 7300 PLUS quantitative PCR instrument.

**Table 1:** Influenza A virus primer and probe sequences.

Primer names	Primer and probe sequences (5’to3’)
H3N2	F: ACCCTCAGTGTGATGGCTTTCAAA R: TAAGGGAGGCATAATCCGGCACAT Probe: ACGAAGCAAAGCCTACAGCAACTGT
H5N1	F: TGGAAAGYGTRAGAAAYGGRACRT R: YRCTARGGAACYCGCCACTG Probe: TAYCCBCASTATTCAGARGAAGC
H7N9	F: AGAAATGAAATGGCTCCTGTCAA R: GGTTTTTTCTTGTATTTTTATATGACTTAG Probe: AGATAATGCTGCATTCCCGCAGATG
H1N1	F: GGGTAGCCCCATTGCAT R: AGAGTGATTCACACTCTGGATTTC Probe: AGAGTGATTCACACTCTGGATTTC

#### 2.2.4 Genome amplification and sequencing

Seventeen random samples of influenza A (H1N1) were selected, and the extracted viral RNA was amplified via the HiScipt II One Step qRT-PCR Kit. The PCR commenced with a 30-min pre-incubation, followed by a denaturation phase at 94°C for three minutes. Forty cycles of amplification were performed under the following conditions: denaturation at 94°C for 30 s, annealing at 55°C for 30 s, extension at 72°C for 2 min, a final 2-min DNA extension at 72°C, and subsequent storage at 4°C. The PCR products were visualized on a 1% agarose gel, utilizing an EB nucleic acid stain, and examined under a UV transilluminator to analyze the electrophoresis results of the PCR products, revealing distinct DNA target bands at the expected locations. Subsequently, the remaining PCR amplification products were sent to Sangon Biotech (Shanghai) Co., Ltd. for bidirectional sequencing using primers provided by the company (refer to Table 2).

**Table 2:** Sequencing primer sequences for influenza A (H1N1) virus.

Amplified fragments	Primer name (5’to3’)
HA	HA-F1: GCAACAAAAATGAAGGCAAT HA-R1: TTCATTCTCCCTTCTTGATC HAR1(1):ATCCAGCCAGCAATGTTACAT HA-F2: TAAAGGGAAAGAAGTCCTCGT HA-R2: CCACTGCTGTGAACTGTG HA-F3: CACAGGATTGAGGAATGT HA-F3(1): GTAAAGCTGGACTCAACAAGGA HA-R3: CATGATTCTGAAATCCTAATG
NA	NA-F1: TAAAATGAATCCAAACCAAAA NA-R1: ACCAAGCGACTGACTCAA NA-R1(1): TCTAGCAGCAGAGTTAGTGTTG NA-F2: TAAGGGAACCATTCATATCAT NA-R2: TAGGGCGTGGATTGT NA-F3: GAACACAAGAGTCTGAATGTGC NA-F3(1): CGAAGAGAACACAATCTGGACT NA-R3: GTCAATGGTGAATGGCAACT

#### 2.2.5 Sequence comparison of influenza a (H1N1) viruses

The DNASTAR V5.0 software was employed for sequence splicing to acquire 17 hemagglutinin (HA) and neuraminidase (NA) gene sequences of influenza A (H1N1) viruses, which were subsequently submitted to GenBank with accession numbers HA (OR517293–OR517309) and NA (OR519614–OR519630), respectively. A total of 22 representative domestic strains from 2019 to 2023 were retrieved from the GISAID and GenBank databases. Within this collection, three strains belonged to branch 6B.1 (EPI_ISL_485562, EPI_ISL_1652533, EPI_ISL_1652534), four strains to branch 6B.1A (EPI_ISL_337378, EPI_ISL_390629, EPI_ISL_410625, EPI_ISL_485409), three strains to branch 6B.1A.5a (EPI_ISL_402310, EPI_ISL_402183, EPI_ISL_402179), four strains to branch 6B.1A.5a.1 (EPI_ISL_410620, EPI_ISL_402276, EPI_ISL_402281, EPI_ISL_485559), two strains to branch 6B.1A.5a.2 (EPI_ISL_528908, EPI_ISL_979818), three strains to branch 6B.1A.5a.2a (EPI_ISL_18068613, EPI_ISL_18068614, EPI_ISL_17465819), three strains to branch 6B.1A.6 (EPI_ISL_485572, EPI_ISL_337348, EPI_ISL_337367), and four strains to branch 6B.2 (EPI_ISL_377079, EPI_ISL_402382, EPI_ISL_1652535, EPI_ISL_1652536). In addition, the World Health Organization-recommended vaccine strains for the 2023–2024 Northern Hemisphere influenza season, A/Wisconsin/67/2022 (H1N1) (OQ203982, OQ203984) and A/Victoria/4897/2022 (H1N1) (OQ718989, OQ718988), were downloaded and served as reference strains. Phylogenetic tree mapping analysis was conducted using the Neighbor-Joining method with Bootstrap values from 1,000 replicates via MEGA software (version 11.0). Furthermore, an analysis of the antigen receptor binding site was performed. The glycosylation sites on the HA and NA genes were predicted online using the NetNGlyc 1.0 Server (http://www.cbs.dtu.dk/services/NetNGlyc/) with the vaccine strain as a reference.

### 2.3 Statistical processing methods

Data compilation and analysis were conducted utilizing SPSS software (version 18.0) statistical software. Categorical data were presented as percentages (%) and comparisons among various groups were executed via the R × C chi-square test. A *p*-value threshold of 0.05 was employed to determine statistical significance.

## 3 Results

### 3.1 Epidemiological characteristics of a (H1N1)

**Figure 1:**
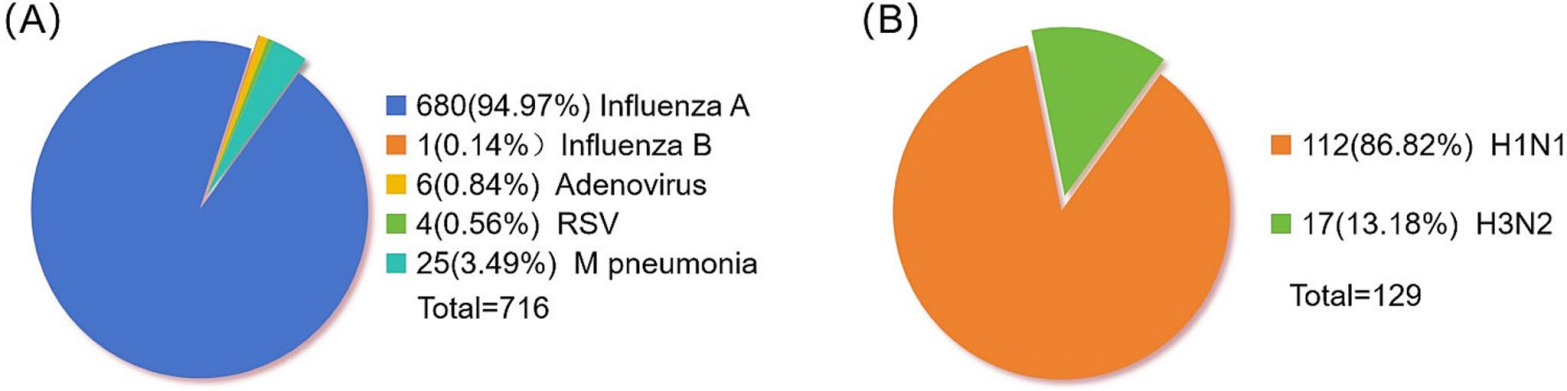
Distribution of positive pathogens in respiratory tract. (A) Statistics of respiratory virus positive samples. (B) Influenza A virus typing.

After examining 2,376 clinical specimens, our analysis yielded 680 specimens positive for influenza A (94.97% of the total), 1 specimen positive for influenza B (0.14%), 6 specimens positive for adenovirus (0.84%), 4 specimens positive for syncytial virus (0.56%), and 25 specimens positive for *Mycoplasma pneumoniae* (Figure 1A). The distribution of influenza A-positive specimens revealed a significant variation across different age groups (Table 3). The age range for the onset of symptoms spanned from 9 months to 99 years, with a median age of 8 years. A chi-square test demonstrated a statistically significant difference in the prevalence of positive infections among the various age categories (*χ*^2^ = 78.50, *p* < 0.05), with the highest incidence rate observed in the 4–6 age bracket at 27.79%. The gender distribution among the positive specimens showed 417 males (61.32%) and 263 females (38.68%), a difference that did not reach statistical significance (*χ*^2^ = 0.025, *p* = 0.874). From the 680 influenza A antigen-positive specimens, a random sample of 129 was further subtyped, revealing 112 cases (86.82%) of H1N1 and 17 cases (13.18%) of H3N2 (Figure 1B).

**Table 3:** Distribution of influenza A antigen positivity by age group, January to March 2023.

Age (years)	Influenza A		*X^2^*	*p-*value
	Positive (number)	Composition ratio (%)		
<1	6	0.88	78.50	<0.05
≥1 ~ 3	67	9.85		
≥4 ~ 6	189	27.79		
≥7 ~ 12	155	22.79		
≥13 ~ 18	46	6.76		
≥19 ~ 30	72	10.59		
≥31 ~ 45	88	12.94		
≥46 ~ 60	37	5.44		
≥61	20	2.94		
Total	680	100.00		

### 3.2 Gene characterization

#### 3.2.1 Homology and phylogenetic analysis

**Figure 2:**
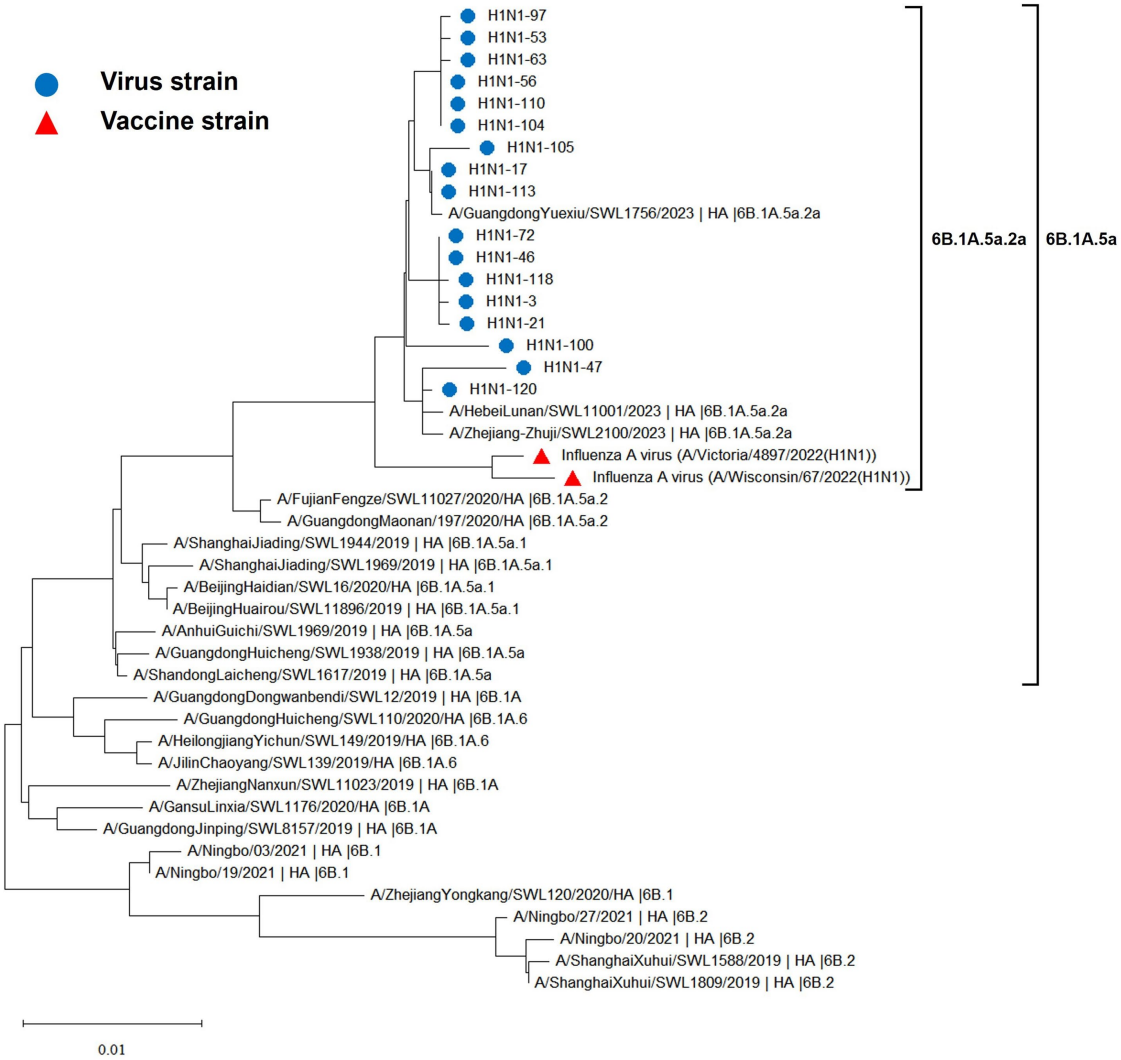
Evolutionary tree diagram of influenza A (H1N1) virus HA genes.

**Figure 3:**
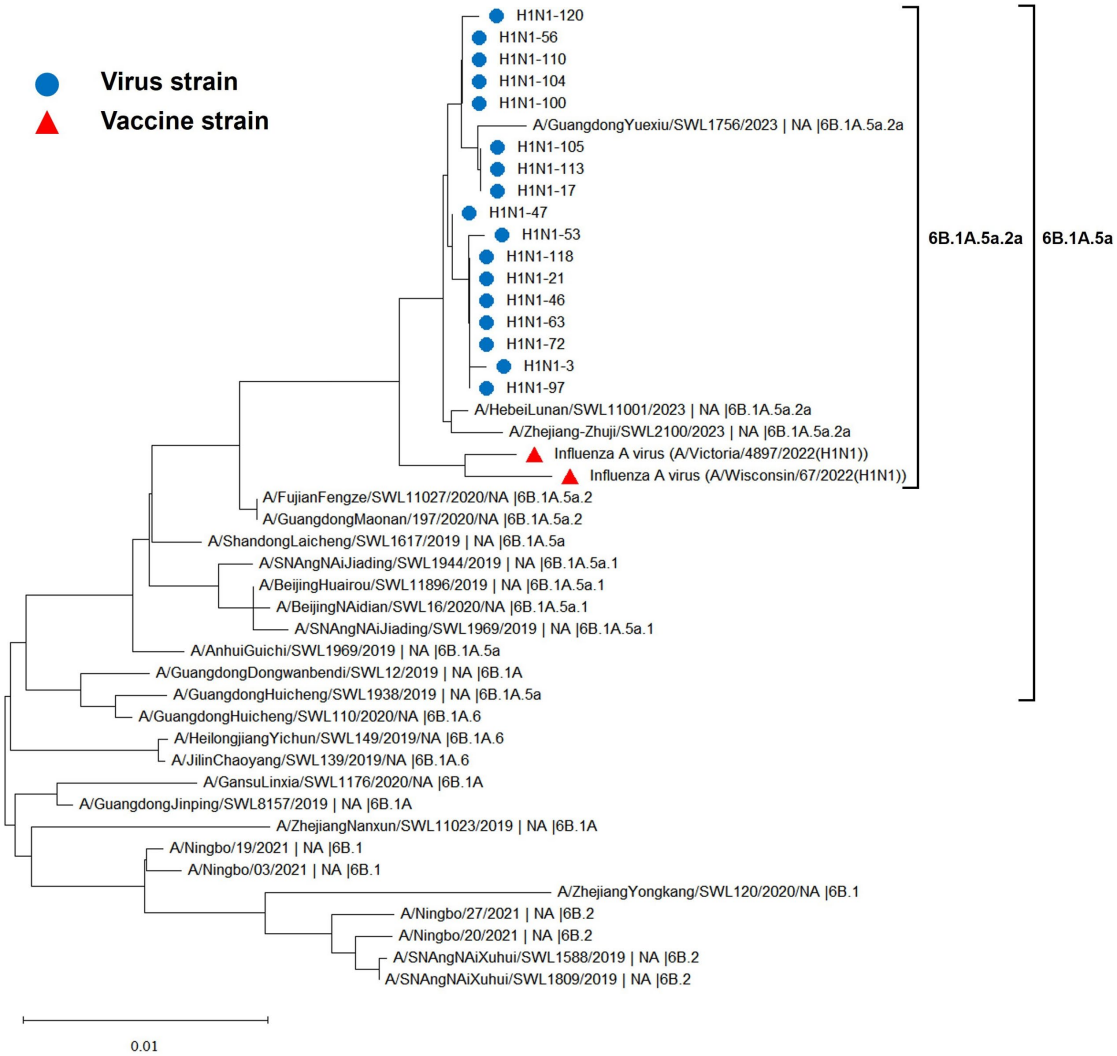
Evolutionary tree diagram of influenza A (H1N1) virus NA genes.

The hemagglutinin (HA) and neuraminidase (NA) genes of the 17 influenza A (H1N1) strains isolated in Hangzhou exhibited a high degree of homology to the vaccine strains, with the extent of evolution appearing to be time-dependent. The amino acid identity between the HA gene of the isolates and the two vaccine strains, A/Wisconsin/67/2022 (H1N1) and A/Victoria/4897/2022 (H1N1), was found to be 98.24 to 98.65% and 98.41 to 98.82%, respectively. Similarly, the amino acid identity between the NA gene and the two vaccine strains was 98.79 to 99.15% and 98.94 to 99.29%, respectively. The phylogenetic analysis of the HA gene (Figure 2) revealed that the H1N1 A strains from 2019 to 2023 remained within branch 6B. Notably, certain domestic representative strains from 2023, the 17 sequenced strains, and the vaccine strains constituted a distinct sub-branch 6B.1A.5a.2a, suggesting a notable evolutionary divergence from the influenza A (H1N1) viruses of 2019 to 2022. The NA gene evolutionary tree (Figure 3) corroborated these findings, indicating that the H1N1 A strains from 2019 to 2023 were located within branch 6B. Similarly, specific domestic representative strains from 2023, the 17 sequenced strains, and the vaccine strains formed a separate sub-branch 6B.1A.5a.2a, demonstrating a significant evolutionary progression when compared to the influenza A (H1N1) viruses from the same period.

#### 3.2.2 Amino acid variant site analysis

##### 3.2.2.1 Analysis of antigenic sites

Molecular analysis of the hemagglutinin (HA) and neuraminidase (NA) antigenic sites of the influenza A (H1N1) virus isolates from Hangzhou was conducted using A/Wisconsin/67/2022 (H1N1) and A/Victoria/4897/2022 (H1N1) as control strains. Seventeen study strains exhibited alterations at a total of 16 amino acid sites in the HA protein, with the S154P mutation present in all 17 strains and the V125M mutation observed in six strains, both associated with the Sa antigenic determinant cluster. A/Hangzhou China/47/2023 strain harbored the N142D and T203N mutations, involved in the Ca antigenic determinants. The HA proteins of the 17 strains underwent mutations at sites 154, 159, 233, 240, 277, 294, 373, and 468, with the remainder being sporadic (refer to Table 4). The NA proteins encoded by these 17 influenza A strains demonstrated mutations at six amino acid sites (13, 50, 200, 339, 382, and 469) (refer to Table 5). It has been demonstrated (15) that the amino acids 140–157 at the N-terminal end of the NA protein are likely to constitute the antigenic determinant cluster, and the 17 strains analyzed exhibited no changes at this critical site.

**Table 4:** Changes in amino acid sites of HA gene of 17 strains of influenza A virus in Hangzhou from January to March 2023.

Amino acid residue	Collection Date	94	125	142	154	159	169	203	233	240	277	294	325	373	416	444	468
OQ203982A/Wisconsin/67/2022		S	V	N	S	R	V	T	T	Q	E	A	R	D	H	V	H
OQ718989A/Victoria/4897/2022		S	V	N	S	R	V	T	A	R	E	A	R	D	H	V	H
A/HangZhou China/03/2023	27/02/2023				P	K			T	Q	D	T		E			N
A/HangZhou China/17/2023	25/02/2023				P	K			T	Q	D	T		E	N		N
A/HangZhou China/21/2023	28/02/2023				P	K			T	Q	D	T		E			N
A/HangZhou China/46/2023	28/02/2023				P	K			T	Q	D	T		E			N
A/HangZhou China/47/2023	28/02/2023			D	P	K		N	T	Q	D	T	K	E			N
A/HangZhou China/53/2023	28/02/2023		M		P	K			T	Q	D	T		E			N
A/HangZhou China/56/2023	26/02/2023		M		P	K			T	Q	D	T		E			N
A/HangZhou China/63/2023	26/02/2023		M		P	K			T	Q	D	T		E			N
A/HangZhou China/72/2023	01/03/2023				P	K			T	Q	D	T		E			N
A/HangZhou China/97/2023	01/03/2023		M		P	K			T	Q	D	T		E			N
A/HangZhou China/100/2023	01/03/2023				P	K	I		T	Q	D	T		E			N
A/HangZhou China/104/2023	01/03/2023		M		P	K			T	Q	D	T		E			N
A/HangZhou China/105/2023	01/03/2023				P	K			T	Q	D	T		E	N		N
A/HangZhou China/110/2023	02/03/2023		M		P	K			T	Q	D	T		E		I	N
A/HangZhou China/113/2023	02/03/2023				P	K			T	Q	D	T		E	N		N
A/HangZhou China/118/2023	02/03/2023	P			P	K			T	Q	D	T		E			N
A/HangZhou China/120/2023	02/03/2023				P	K			T	Q	D	T		E			N

**Table 5:** Changes in amino acid sites of NA gene of 17 influenza A virus strains in Hangzhou from January to March 2023.

Amino Acid Residue	Collection Date	13	50	200	339	382	469
OQ203982A/Wisconsin/67/2022		V	N	S	L	G	N
OQ718989A/Victoria/4897/2022		I	D	S	S	E	N
A/HangZhou China/03/2023	27/02/2023	I	N	N	S	G	K
A/HangZhou China/17/2023	25/02/2023	I	N	N	S	G	K
A/HangZhou China/21/2023	28/02/2023	I	N	N	S	G	K
A/HangZhou China/46/2023	28/02/2023	I	N	N	S	G	K
A/HangZhou China/47/2023	28/02/2023	I	N	N	S	G	K
A/HangZhou China/53/2023	28/02/2023	I	N	N	S	G	K
A/HangZhou China/56/2023	26/02/2023	I	N	N	S	G	K
A/HangZhou China/63/2023	26/02/2023	I	N	N	S	G	K
A/HangZhou China/72/2023	01/03/2023	I	N	N	S	G	K
A/HangZhou China/97/2023	01/03/2023	I	N	N	S	G	K
A/HangZhou China/100/2023	01/03/2023	I	N	N	S	G	K
A/HangZhou China/104/2023	01/03/2023	I	N	N	S	G	K
A/HangZhou China/105/2023	01/03/2023	I	N	N	S	G	K
A/HangZhou China/110/2023	02/03/2023	I	N	N	S	G	K
A/HangZhou China/113/2023	02/03/2023	I	N	N	S	G	K
A/HangZhou China/118/2023	02/03/2023	I	N	N	S	G	K
A/HangZhou China/120/2023	02/03/2023	I	N	N	S	G	K

##### 3.2.2.2 Key site analysis

The 17 strains under investigation maintained a high degree of conservation in the receptor-binding sites of the 220 loop (residues 218–225), the 130 loop (residues 132–135), and the 190 loop (residues 187–195), as well as at the base of the pocket with Y91, W150, H180, and Y192. Notably, nine critical amino acid sites (16) associated with resistance to ceramidase inhibitors (E119, Q136, Y155, I223, S247, H275, R293, N295, and Q313) remained unmutated. Despite recent evidence indicating that the H275Y mutation confers resistance to Tamiflu (17–19), this resistance-conferring site was not observed in the current study. It is a widely recognized principle that the hemagglutinin (HA) cleavage site exhibits low pathogenicity when composed of a single basic amino acid, and it demonstrates high pathogenicity when it contains at least four consecutive basic amino acids (20). The amino acid sequence of the HA cleavage site for the 17 strains of A(H1N1) virus in this study was PSIQSR↓GLF, with a single basic amino acid, R (position 327), bridging the HA1 and HA2 subunits, and no sequence of consecutive basic amino acids was detected in the vicinity of the cleavage site.

##### 3.2.2.3 Analysis of glycosylation sites

Studies have confirmed the high conservation of potential glycosylation sites at positions 28, 40, 104, 304, 498, and 557 on the hemagglutinin (HA) protein across all H1N1 strains isolated from both animal and human sources (21). However, glycosylation sites at positions 142, 172, 177, and 179 on the HA head, and sites at positions 71 and 286 on the HA stem, typically manifest only during particular evolutionary phases of human seasonal influenza. The NetNGlyc 1.0 Server online tool predicted a total of seven HA glycosylation sites from 17 strains as 28, 40, 104, 179, 304, 498, and 557. Additionally, eight NA glycosylation sites were determined, including glycosylation sites 42, 50, 58, 63, 68, 88, 146, and 235.

## 4 Discussion

Influenza represents a significant public health concern as an acute respiratory infection caused by the influenza virus, annually manifesting as seasonal outbreaks with a peak during the winter and spring months. The disease has a propensity to escalate during periods of increased population aggregation, such as in educational facilities, childcare centers, and geriatric care facilities (22). National influenza sentinel surveillance data show that influenza in the years 2012 ~ 2019 showed obvious seasonal epidemic characteristics, with an average annual positive influenza virus test rate of 14.57%. After the outbreak of COVID-19, the regular seasonal pattern of influenza in China was broken, with a shallow epidemic level in FY2020 ~ 2021 (average annual positive test rate < 1.0%) and a resurgence of the winter peak in FY2022 (23). While the general populace is vulnerable to influenza infections, there exists a variability in susceptibility among different age cohorts, with children exhibiting a greater incidence rate compared to adults. An analysis of influenza incidence in unsusceptible populations from a meta-analysis of 32 randomized controlled trials assessing influenza vaccination across the globe (24) indicated that the incidence of symptomatic influenza was 12.7% among pediatric individuals (<18 years), 4.4% in the adult demographic, and 7.2% among the older adult (≥65 years). The study elucidated a demographic disparity in the prevalence of influenza A, documenting a higher incidence among pediatric populations compared to adults, yet a decreased prevalence in the older adult, which contradicted prior research findings. The infection rates for the 4 ~ 6 and 7 ~ 12 age brackets collectively represented a substantial 50.58% of the total infections, a figure suggestive of a correlation with the frequent influenza A outbreaks reported in primary and secondary educational settings during the initial months of 2023. The observed higher incidence rates within the 19 ~ 30 and 31 ~ 45 age groups may be attributable to the propensity of this demographic to engage in frequent external activities and maintain extensive community contact. Furthermore, the accelerating pace of life and heightened competitive pressures experienced by young individuals could engender physiological and psychological stress, potentially compromising immune function. The amalgamation of late nights and irregular eating habits further exacerbates this vulnerability, making this demographic more susceptible to viral challenges. This finding underscores the necessity for young adults to prioritize physical health and immune enhancement strategies. Conversely, the infection rate among individuals aged 61 and above was significantly lower, at 2.94%, possibly due to the older adult’s reduced propensity to seek medical attention for fever-related symptoms, thereby potentially skewing the data away from the true incidence rate.

The evolutionary trajectory of influenza viruses is predominantly guided by two pivotal mechanisms: amino acid site mutations and reassortments among influenza subtypes and internal genes (25). These mechanisms can engender antigenic drift and antigenic shift, respectively. The A/H1N1pdm virus emerged via a complex triple recombination event and infected humans in 2009, promptly diffusing within the human population and replaced the prior seasonal H1N1 influenza strains as the predominant seasonal influenza virus, thereby posing significant threats and exerting a considerable burden on global public health. Annual influenza vaccination has emerged as the most cost-effective preventive measure against influenza. However, the influenza vaccine is not yet integrated into the national immunization program, and its coverage rate in China remains low. According to the U.S. Centers for Disease Control and Prevention (CDC), a total of 175 million doses of influenza vaccine were distributed in the U.S. during the 2019–2020 flu season, with an adult vaccination rate of approximately 48.4%. The China Institute for Food and Drug Control (CIFDC) reports that the amount of influenza vaccine approved and distributed in China in 2020 was 0.58 billion doses, and the national vaccination rate was less than 4.2%, which is less than one-tenth of that in the U.S. Currently, the influenza vaccine has not garnered widespread attention. Related studies have indicated that the protection rate of existing vaccines is only 40–60% (26), and their effectiveness will be further diminished against new influenza strains that do not match the vaccine strains. In this study, the homology between the HA gene of 17 virus strains and the amino acids of two vaccine strains, A/Wisconsin/67/2022 (H1N1) and A/Victoria/4897/2022 (H1N1), is 98.24 to 98.65% and 98.41 to 98.82%, respectively. The amino acid sequence homology between the neuraminidase (NA) gene of the influenza A/H1N1pdm09 virus and the two vaccine strains is 98.79 to 99.15% and 98.94 to 99.29%, respectively. Genetic evolutionary analysis indicates that influenza strains prevalent from 2019 to 2023 belonged to 6B.1 and 6B.2, which evolved continuously into 6B.1A, 6B.1A.5, 6B.1A.5a, and 6B.1A.5a.2a, based on a specific evolutionary cluster of mutation sites. The 17 strains, the vaccine strain, and some of the 2023 representative strains are in the same branch and have independently formed the 6B.1A.5a.2a evolutionary branch. This suggests that the A/H1N1pdm09 influenza strain circulating in Hangzhou in 2023 and the World Health Organization (WHO)-recommended vaccine strains for the 2023–2024 season exhibit high homology and favorable compatibility. Vaccination with the recommended vaccine strains can provide enhanced protection against the current circulating A/H1N1pdm09 influenza strain. This finding is discrepant from the aforementioned related studies, as the vaccine efficacy is not solely dependent on the homology between the vaccine strain and the isolate but also influenced by the immune status of the vaccinated population, vaccination protocols, and the timing of vaccination. It has been shown that the appearance of mutation sites N156K, L161I, A186T, and Q189E has led to the evolution of 6B.1A.5a.2 into the 6B.1A.5a.2a branch, in which the N156K mutation in the Sa antigenic epitope significantly reduces the recognition of vaccine sera, which leads to immune escape and increased susceptibility in the population. Therefore, it is crucial to keep abreast of the evolutionary trends of virus epidemics and continuous antigenic monitoring and timely updating of vaccines (27).

It is widely acknowledged that the emergence of a novel antigenic variant with significant epidemiological implications necessitates alterations at four or more amino acid positions within the HA1 domain of the protein. These alterations must pertain to more than two antigenic determinant cluster sites (28). The HA protein harbors four antigenic determinants within its structure, specifically: Sa (amino acids 124 ~ 125, 153 ~ 157, 159 ~ 164); Sb (184 ~ 195); Ca (137 ~ 142, 166 ~ 170, 203 ~ 205, 221 ~ 222, 235 ~ 237); and Cb (70 ~ 75). In the current study, we identified a total of 16 amino acid site changes within the HA protein across 17 strains. The S154P mutation was universally present in all 17 strains, whereas the V125M mutation was detected in only six strains, both of which are associated with the Sa antigenic determinant cluster. Moreover, strain A/Hangzhou China/47/2023 exhibited the N142D and T203N mutations, which are implicated in the Ca antigenic determinants. Seventeen hemagglutinin (HA) proteins were subject to mutations at specific amino acid positions, including sites 154, 159, 233, 240, 277, 294, 373, and 468, whereas variants at other sites were sporadic. The neuraminidase (NA) proteins encoded by seventeen influenza A strains exhibited mutations at six amino acid residues: 13, 50, 200, 339, 382, and 469. These findings did not fulfill the criteria for the emergence of novel antigenic variants with significant epidemiological implications. However, an increased incidence of mutations at amino acid sites was observed. It has been documented that the majority of H1N1 epidemic strains have manifested the S203T mutation since 2011 (29). This study revealed the presence of not only this mutation but also the T203N mutation at position 203, which is involved in the Ca antigenic determinants. Whether this specific amino acid site serves as a foundation for antigenic variation requires further investigation. Nonetheless, if this point mutation accumulates, it could lead to a reconfiguration of the antigenic site, thereby potentially escaping the population-specific immune barrier and posing a pandemic threat. It has been shown that the nuclear protein NP of influenza A virus inhibits the host innate immune response by inducing mitochondrial autophagy leading to the degradation of the mitochondrial anchoring protein MAVS, thereby blocking MAVS-mediated antiviral signaling and promoting viral replication (30). Professor Zhou Hongbo’s team also revealed a new mechanism of influenza virus immune escape, i.e., PB1-F2 protein, as an important virulence factor of influenza virus, can escape from the antiviral natural immune response by inhibiting the production of type I interferon (31). Therefore, it is necessary for us to closely monitor the antigenic variation of the influenza virus and to understand the ways of influenza virus escapes the host’s natural immune response, which will help to develop antiviral therapeutic strategies and provide new ideas to block the influenza virus infection.

The amino acid residues within the pocket of the hemagglutinin (HA) receptor-binding site (RBS) are critical in determining the specificity of human influenza virus receptor recognition. The RBS exhibits considerable conservation, such that alterations in a single amino acid can significantly influence the binding specificity of the virus to its host and, consequently, the pathogenicity of the influenza virus. Internationally, there is a consensus that mutations at position 222, within loop 220 (e.g., D222E, D222N, D222G), lead to HA proteins with enhanced affinity for the SAα2,3Gal receptor. This receptor is predominantly expressed in the lower respiratory tract, and its interaction with the mutated HA proteins is associated with more severe lower respiratory tract infections (32, 33). In the strains examined in this study, no such mutations were detected; However, all 17 strains harbored an arginine (R) at position 222, which contrasts with previous reports in the literature that described all aspartic acid (D) at this position, and there is a lack of relevant literature documenting the D222R mutation. Continuous monitoring of this site is essential to elucidate its role in antigenic variation and to determine whether it possesses particular research significance. The amino acid sequence analysis of the hemagglutinin (HA) cleavage site from 17 strains of the A(H1N1) virus within this investigation revealed a configuration of PSIQSR↓GLF. Notably, a single basic amino acid, arginine (R), bridged the HA1 and HA2 subunits, devoid of any proximal consecutive basic amino acids characteristic indicative of low pathogenicity influenza viruses, as previously reported (34). This feature aligns with the clinical presentation of the selected patient specimens, all of which exhibited mild illness. The neuraminidase (NA) enzyme constitutes the target for anti-influenza therapeutics, with its catalytic active site pivotal in the design of drugs that inhibit influenza A virus propagation. Mutations within the NA gene are pivotal in the development of resistance against neuraminidase inhibitors, with nine critical amino acid positions identified in this context. None of the 17 strains under study exhibited mutations at these resistance-associated sites, suggesting a lack of resistance to neuraminidase inhibitors. In recent years, an increasing body of research has elucidated that the H275Y mutation conveys resistance to oseltamivir. Our study did not detect any mutations at the resistance locus, suggesting that the virus maintains susceptibility to this antiviral agent. And some studies have shown that the S247N mutation reduces its sensitivity to zanamivir. With the widespread use of NAIs, the current global rate of resistance to NAIs is up to 1.9%: studies in the European region have found that the rate of resistance to influenza A (H1N1) viruses has risen from 0.4% (2014 ~ 2015 pandemic seasons) to 0.9% (2015 ~ 2016 pandemic seasons) and 1.9% (2017 ~ 2018 pandemic seasons), with an overall prevalence of resistant influenza strains ranging from 0.3 to 0.9% (35). Nevertheless, ongoing surveillance of resistance mutations is imperative to avert future influenza pandemics.

Throughout evolution, viruses have the capacity to modify or acquire glycosylation sites, which, though not essential for viral persistence, can obscure antigenic determinants and enzymatic cleavage sites, facilitating evasion of host immune responses and aiding in the emergence of drug-resistant variants. Moreover, glycosylation can modulate the hydrolytic activity of the hemagglutinin (HA) protein, influencing the pathogenicity of influenza viruses (36). In the current study, we analyzed a total of seven HA and eight neuraminidase (NA) glycosylation sites across 17 influenza virus strains. The HA glycosylation sites have typically been well-conserved; however, the appearance of the 179th glycosylation site has been observed exclusively during particular evolutionary phases of human seasonal influenza. Whether the presence of this site in our sample signifies entry into a distinct evolutionary phase of human seasonal influenza requires additional investigation, which should include an enlarged sample size, broader regional representation, and continued surveillance. Nonetheless, the emergence of this glycosylation site might also be correlated with the increased incidence of national influenza A cases in early 2023 and the widespread outbreak of novel coronavirus infections in late 2022 and early 2023. This is significant because research has suggested that influenza A viruses can potentiate the infectivity of novel coronaviruses by facilitating cell entry and increasing viral load. Consequently, we must remain vigilant to the risks associated with co-infection of influenza A (H1N1) virus with the novel coronavirus.

In summary, influenza A (H1N1) isolates from Hangzhou, Hangzhou City, exhibited a high degree of homology with the vaccine strains, indicating that the vaccine was effective in immunoprotection for the population. Notwithstanding, this study has several limitations, most notably a small sample size, which encompassed only 17 influenza A (H1N1) isolates for the sequencing and analysis of the hemagglutinin (HA) and neuraminidase (NA) genes. This constraint may have compromised the generalizability of the findings. Moreover, there is an imperative to augment surveillance efforts. Additionally, although the World Health Organization (WHO)-recommended influenza vaccine for the 2023–2024 season is efficacious, we must remain vigilant in influenza surveillance due to the ongoing evolution and mutation of the virus strains. Continuous monitoring of drug resistance mutations, key binding sites, glycosylation sites, and other critical aspects is crucial, allowing for prompt modifications to antiviral medications and vaccination strategies. Should influenza viruses persist in mutating and generating novel strains with pandemic potential, it could precipitate another global influenza pandemic, posing a significant threat to public health.

## Data Availability

The datasets presented in this study can be found in online repositories. The names of the repository/repositories and accession number(s) can be found in the article/supplementary material.

## References

[1] Bouvier NM Please P. The biology of influenza viruses. Vaccine. (2008) 26:D49–53. doi: 10.1016/j.vaccine.2008.07.039, PMID: 19230160 PMC3074182

[2] Cui F Luo H Zhou L Yin D Zheng C Wang D. Transmission of pandemic influenza a (H1N1) virus in a train in China. Epidemiology. (2011) 21:271–7. doi: 10.2188/jea.je20100119, PMID: 21646746 PMC3899419

[3] Jain R Goldman RD. Novel influenza A(H1N1): clinical presentation, diagnosis, and management. Pediatr Emerg Care. (2009) 25:791–6. doi: 10.1097/PEC.0b013e3181c3c8f8 19915434

[4] World Health Organization. Global influenza strategy 2019–2030. World Health Organization. (2019). Available at: https://iris.who.int/handle/10665/311184.

[5] Lampejo T. Influenza and antiviral resistance: an overview. Eur J Clin Microbiol Infect Dis. (2020) 39:1201–8. doi: 10.1007/s10096-020-03840-9 32056049 PMC7223162

[6] Iuliano AD Roguski KM Chang HH Muscatello DJ Palekar R Tempia S. Estimates of global seasonal influenza-associated respiratory mortality: a modelling study. Lancet. (2018) 391:1285–300. doi: 10.1016/S0140-6736(17)33293-2, PMID: 29248255 PMC5935243

[7] Linster M Schrauwen EJA van der Vliet S Burke DF Lexmond P Bestebroer TM. The molecular basis for antigenic drift of human a/H2N2 influenza viruses. ASM J CD. (2019) 93:e01907–18. doi: 10.1128/JVI.01907-18, PMID: 30700609 PMC6450109

[8] Virk RK Jayakumar J Mendenhall IH Moorthy M Lam P Linster M. Divergent evolutionary trajectories of influenza B viruses underlie their contemporaneous epidemic activity. Proc Natl Acad Sci USA. (2020) 117:619–28. doi: 10.1073/pnas.1916585116, PMID: 31843889 PMC6955377

[9] Gao J Couzens L Burke DF Wan H Wilson P Memoli MJ. Antigenic drift of the influenza A(H1N1) pdm09 virus neuraminidase results in reduced effectiveness of a/California/7/2009 (H1N1pdm09)-specific antibodies. MBio. (2019) 10:e00307–19. doi: 10.1128/mBio.00307-19, PMID: 30967460 PMC6456748

[10] Chen N Wang R Zhu W Hao X Wang J Chen G. Development and characterization of an antibody that recognizes influenza virus N1 neuraminidases. PLoS One. (2024) 19:e0302865. doi: 10.1371/journal.pone.0302865, PMID: 38723016 PMC11081314

[11] Lloren KKS Kwon JJ Choi WS Jeong JH Ahn SJ Choi YK. In vitro and in vivo characterization of novel neuraminidase substitutions in influenza A(H1N1) pdm09 virus identified using Laninamivir-mediated in vitro selection. Virol. (2019) 93:e01825–18. doi: 10.1128/JVI.01825-18, PMID: 30602610 PMC6401420

[12] Świerczyńska M Mirowska-Guzel DM Pindelska E. Antiviral Drugs in Influenza. Int J Environ Res Public Health. (2022) 19:3018. doi: 10.3390/ijerph19053018, PMID: 35270708 PMC8910682

[13] Dong G Peng C Luo J Wang C Han L Wu B. Adamantane-resistant influenza a viruses in the world (1902-2013): frequency and distribution of M2 gene mutations. PLoS One. (2015) 10:e0119115. doi: 10.1371/journal.pone.0119115, PMID: 25768797 PMC4358984

[14] Samson M Pizzorno A Abed Y Boivin G. Influenza virus resistance to neuraminidase inhibitors. Antivir Res. (2013) 98:174–85. doi: 10.1016/j.antiviral.2013.03.014 23523943

[15] Russell RJ Haire LF Stevens DJ Collins PJ Lin YP Blackburn GM. The structure of H5N1 avian influenza neuraminidase suggests new opportunities for drug design. Nature. (2006) 443:45–9. doi: 10.1038/nature05114, PMID: 16915235

[16] McKimm-Breschkin JL. Influenza neuraminidase inhibitors: antiviral action and mechanisms of resistance. Influenza Other Respir Viruses. (2013) 7:25–36. doi: 10.1111/irv.12047, PMID: 23279894 PMC4942987

[17] Gubareva LV Besselaar TG Daniels RS Fry A Gregory V Huang W. Global update on the susceptibility of human influenza viruses to neuraminidase inhibitors, 2015-2016. Antivir Res. (2017) 146:12–20. doi: 10.1016/j.antiviral.2017.08.004, PMID: 28802866 PMC5667636

[18] Sheu TG Deyde VM Okomo-Adhiambo M Garten RJ Xu X Bright RA. Surveillance for neuraminidase inhibitor resistance among human influenza a and B viruses circulating worldwide from 2004 to 2008. Antimicrob Agents Chemother. (2008) 52:3284–92. doi: 10.1128/AAC.00555-08, PMID: 18625765 PMC2533500

[19] Renaud C Kuypers J Englund JA. Emerging oseltamivir resistance in seasonal and pandemic influenza a/H1N1. Clin Virol. (2011) 52:70–8. doi: 10.1016/j.jcv.2011.05.019 21684202

[20] Ke C Mok CKP Zhu W Zhou H He J Guan W. Human infection with highly pathogenic avian influenza A(H7N9) virus, China. Emerg Infect Dis. (2017) 23:1332–40. doi: 10.3201/eid2308.170600, PMID: 28580899 PMC5547808

[21] Sun S Wang Q Zhao F Chen W Li Z. Glycosylation site alteration in the evolution of influenza a (H1N1) viruses. PLoS One. (2011) 6:e22844. doi: 10.1371/journal.pone.0022844, PMID: 21829533 PMC3145772

[22] Finnie TJ Copley VR Hall IM Leach S. An analysis of influenza outbreaks in institutions and enclosed societies. Epidemiol Infect. (2014) 142:107–13. doi: 10.1017/S0950268813000733, PMID: 23570654 PMC3857146

[23] Huang WJ Cheng YH Tan MJ Liu J Li XY Zeng XX. Epidemiological and virological surveillance of influenza viruses in China during 2020-2021. Infect Dis Poverty. (2022) 11:74. doi: 10.1186/s40249-022-01002-x, PMID: 35768826 PMC9244124

[24] Somes MP Turner RM Dwyer LJ Newall AT. Estimating the annual attack rate of seasonal influenza among unvaccinated individuals: a systematic review and meta-analysis. Vaccine. (2018) 36:3199–207. doi: 10.1016/j.vaccine.2018.04.063, PMID: 29716771

[25] Shao W Li X Goraya MU Wang S Chen JL. Evolution of influenza a virus by mutation and re-assortment. Int J Mol Sci. (2017) 18:1650. doi: 10.3390/ijms18081650, PMID: 28783091 PMC5578040

[26] Li J Zhang Y Zhang X Liu L. Influenza and universal vaccine research in China. Viruses. (2022) 15:116. doi: 10.3390/v15010116, PMID: 36680158 PMC9861666

[27] Strengell M Ikonen N Ziegler T Julkunen I. Minor changes in the hemagglutinin of influenza A(H1N1)2009 virus alter its antigenic properties. PLoS One. (2011) 6:e25848. doi: 10.1371/journal.pone.0025848, PMID: 22022458 PMC3191144

[28] Xu R Ekiert DC Krause JC Hai R Crowe JE Jr Wilson IA. Structural basis of preexisting immunity to the 2009 H1N1 pandemic influenza virus. Science. (2010) 328:357–60. doi: 10.1126/science.1186430, PMID: 20339031 PMC2897825

[29] Zolotarova O Budzanivska I Leibenko L Radchenko L Mironenko A. Antigenic site variation in the hemagglutinin of pandemic influenza A(H1N1) pdm09 viruses between 2009-2017 in Ukraine. Pathogens. (2019) 8:194. doi: 10.3390/pathogens8040194, PMID: 31635227 PMC6963832

[30] Zhang B Xu S Liu M Wei Y Wang Q Shen W. The nucleoprotein of influenza a virus inhibits the innate immune response by inducing mitophagy. Autophagy. (2023) 19:1916–33. doi: 10.1080/15548627.2022.2162798, PMID: 36588386 PMC10283423

[31] Wang R Zhu Y Ren C Yang S Tian S Zhou H. Influenza a virus protein PB1-F2 impairs innate immunity by inducing mitophagy. Autophagy. (2020) 17:496–511. doi: 10.1080/15548627.2020.1725375, PMID: 32013669 PMC8007153

[32] Ruggiero T De Rosa F Cerutti F Pagani N Allice T Stella ML. A(H1N1) pdm09 hemagglutinin D222G and D222N variants are frequently harbored by patients requiring extracorporeal membrane oxygenation and advanced respiratory assistance for severe A(H1N1) pdm09 infection. Influenza Other Respir Viruses. (2013) 7:1416–26. doi: 10.1111/irv.12146, PMID: 23927713 PMC4634302

[33] Baldanti F Campanini G Piralla A Rovida F Braschi A Mojoli F. Severe outcome of influenza a/H1N1/09v infection associated with 222G/N polymorphisms in the haemagglutinin: a multicentre study. Clin Microbiol Infect. (2011) 17:1166–9. doi: 10.1111/j.1469-0691.2010.03403.x, PMID: 20946414

[34] Peiris JS de Jong MD Guan Y. Avian influenza virus (H5N1): a threat to human health. Clin Microbiol Rev. (2007) 20:243–67. doi: 10.1128/CMR.00037-06, PMID: 17428885 PMC1865597

[35] Bragstad K Hungnes O Litleskare I Nyrerød HC Dorenberg DH Hauge SH. Community spread and late season increased incidence of oseltamivir-resistant influenza A(H1N1) viruses in Norway 2016. Influenza Other Respir Viruses. (2019) 13:372–81. doi: 10.1111/irv.12637, PMID: 30834715 PMC6586177

[36] Schulze IT. Effects of glycosylation on the properties and functions of influenza virus hemagglutinin. J Infect Dis. (1997) 176:S24–8. doi: 10.1086/514170 9240690

